# Case Report: Cranial intradural chordoma following extradural spinal chordoma

**DOI:** 10.3389/fsurg.2025.1598308

**Published:** 2025-10-01

**Authors:** Ramazan Sarı, Mehmet Osman Akçakaya, Kadir Özyılmaz, Yasin Temel, Ilhan Elmacı

**Affiliations:** 1 Department of Neurosurgery, Demiroglu Bilim University, Istanbul, Türkiye; 2 Department of Anesthesiology, Demiroglu Bilim University, Istanbul, Türkiye; 3 Department of Neurosurgery, Maastricht University Medical Center, Maastricht, Netherlands; 4 Elmacı Neurosurgery Institute, Istanbul, Türkiye

**Keywords:** intradural chordoma, cranial chordoma, metastasis, brain parenchymal lesion, spinal chordoma

## Abstract

Chordomas are rare, locally invasive, and slow-growing neoplasms that originate from remnants of the primitive notochord. They account for approximately 1% of all intracranial tumors and are typically found in the sacrococcygeal region or at the skull base. Purely intradural intracranial chordomas are exceptionally rare, with only 67 cases documented to date, to the best of our knowledge. These tumors are generally situated near the midline. We present the case of a 67-year-old male who developed hemiparesis and hemihypoesthesia 6 years after undergoing surgery for a classical spinal chordoma that was purely extradural. Magnetic resonance imaging revealed a mass in the frontoparietal area, initially suggestive of an intraaxial tumor. However, intraoperative findings indicated that the lesion was extraaxial, and histopathological evaluation confirmed it as an intradural chordoma. To our knowledge, this is the first reported case of a chordoma within the brain parenchyma and away from the midline.

## Introduction

Chordomas are rare, locally invasive, and slow-growing tumors that develop from remnants of the primitive notochord and are typically found along the axial skeleton (1, 2). They account for approximately 1% of intracranial tumors and 1%–4% of all primary bone tumors (3–5). Approximately 50% of chordomas occur in the sacrococcygeal region, 35% at the skull base, and 15% in the mobile spine (6–8). Although histopathologically classified as benign, chordomas can extend beyond the dura and cause significant bone destruction, making complete surgical excision difficult (2, 4). Even after total macroscopic removal, recurrence is frequent (9).

Purely intradural chordomas without involvement of the dura or bone are extremely rare. These tumors are most often located along the midline, particularly in the prepontine cistern and the supra- or intrasellar regions (1). Compared with conventional chordomas, intradural chordomas are considered to have a more favorable prognosis, as their surgical removal is reportedly easier due to the clear boundaries of the lesions (3, 6). Spinal chordomas may also arise as metastatic seeding following surgical intervention for cranial chordomas (7, 8). In this report, we present a case involving a purely extradural spinal chordoma that was surgically treated twice, with the patient developing a cranial intradural chordoma 3 years after the second procedure. A fluorodeoxyglucose-positron emission tomography/computed tomography (FDG-PET/CT) scan performed at the time of the initial presentation revealed no such intracranial lesion. To the best of our knowledge, this is the first reported case of an intradural chordoma located within the frontoparietal parenchyma and away from the midline.

## Case report

**Figure 1:**
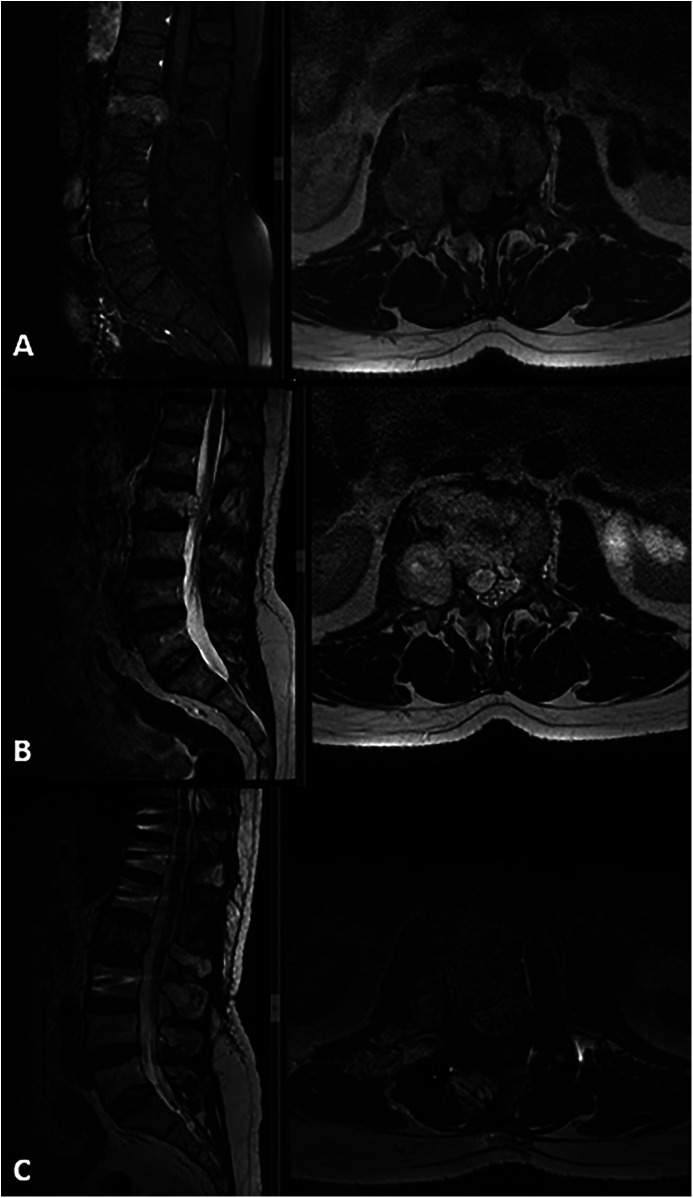
Preoperative MRI: **(A)** T1-weighted sagittal and axial MRI with contrast, **(B)** T2-weighted sagittal and axial MRI revealed a mass lesion involving the right pedicle and body of the L2 vertebra. There was approximately 50% height loss of the vertebral body at that level, with partial tumor extension into the spinal canal causing compression of the thecal sac from the right side. The lesion demonstrated diffuse contrast enhancement and appeared hyperintense on T2-weighted images. **(C)** Postoperative T2-weighted sagittal and axial images showed a total laminectomy at L1 and right hemipartial laminectomy at L2, subtotal tumor removal, and decompression. Note the use of unilateral (left) pedicle screws at the L2 level.

**Figure 2:**
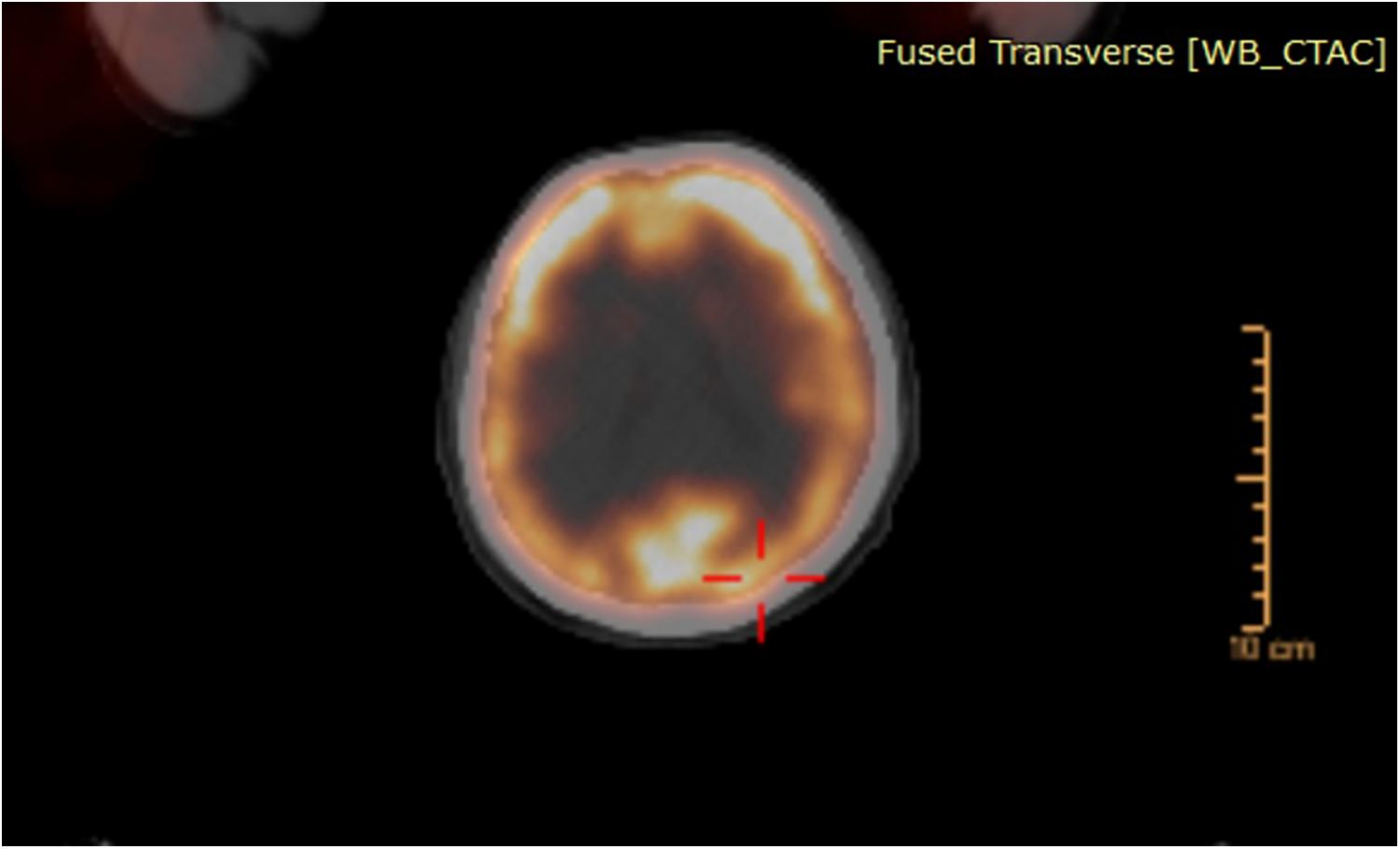
FDG-PET/CT scan demonstrating no intracranial lesions with increased uptake or causing any mass effect.

A 67-year-old male patient presented to our outpatient clinic with a complaint of low back pain that had begun 6 months prior. During the past month, the pain had started to radiate into his left leg. Neurological examination revealed muscle strength of 4/5 in the left tibialis anterior and extensor hallucis longus, along with localized tenderness upon palpation over the L2 vertebra. Magnetic resonance imaging (MRI) revealed a mass involving the right pedicle and body of the L2 vertebra. There was a 50% reduction in vertebral body height at this level, with partial tumor extension into the spinal canal and compression of the thecal sac from the right side (Figures 1A,B). The lesion appeared hypointense on T1-weighted images and hyperintense on T2-weighted images and exhibited diffuse contrast enhancement. An FDG-PET/CT was also performed, which demonstrated increased uptake at the L2 vertebra (SUVmax, 12.5), suggestive of metastasis. No intracranial lesions exhibited increased uptake or produced mass effect on the FDG-PET/CT (Figure 2). Based on these findings, the lesion was initially considered a potential metastatic tumor, and surgical intervention was scheduled. Surgery was performed via a posterior approach. A total laminectomy at L1 and right-sided hemipartial laminectomy at L2 were conducted to achieve subtotal decompression and tumor removal. Posterior stabilization from T11 to L4 was achieved using bilateral pedicle screws, with unilateral placement at the L2 level on the left side (Figure 1C). The lesion was entirely extradural. Histopathological examination revealed nodular tumor tissue within a rich vascular background, characterized by focal, prominent epithelial proliferation. The tumor cells were predominantly clear, partially physaliphorous, and partially eosinophilic with abundant cytoplasm, showing low-grade atypia. While mucin was present in small areas and there were single-layered cell patterns resembling adenoid structures, well-formed glandular architecture was not identified. Immunohistochemically, the tumor was strongly positive for vimentin, CDX2, TTF-1 (in 25% of cells), epithelial membrane antigen (EMA), and Pan-CK (5/6/8/18). It was negative for S-100, CK-20, CK-7, CK-5/6, CD10, and renal cell carcinoma (RCC). Brachyury staining was not performed. Due to the absence of S-100 positivity, a diagnosis of chordoma was ruled out. Although CDX2 positivity indicated intestinal differentiation, the lack of CK7 and CK20 expression, which are typically seen in gastrointestinal tumors, argued against that origin. Possible primary sources considered included adrenal, hepatic, and prostatic carcinomas. A definitive diagnosis could not be established, and the lesion was interpreted as a metastasis of clear cell carcinoma from an unknown primary. The postoperative course was uneventful, his symptoms were resolved, and the postoperative neurological examination of the patient was unremarkable. Stereotactic radiosurgery (TruBeam) was administered, delivering a total dose of 30 cGy in 10 fractions to the tumor site.

**Figure 3:**
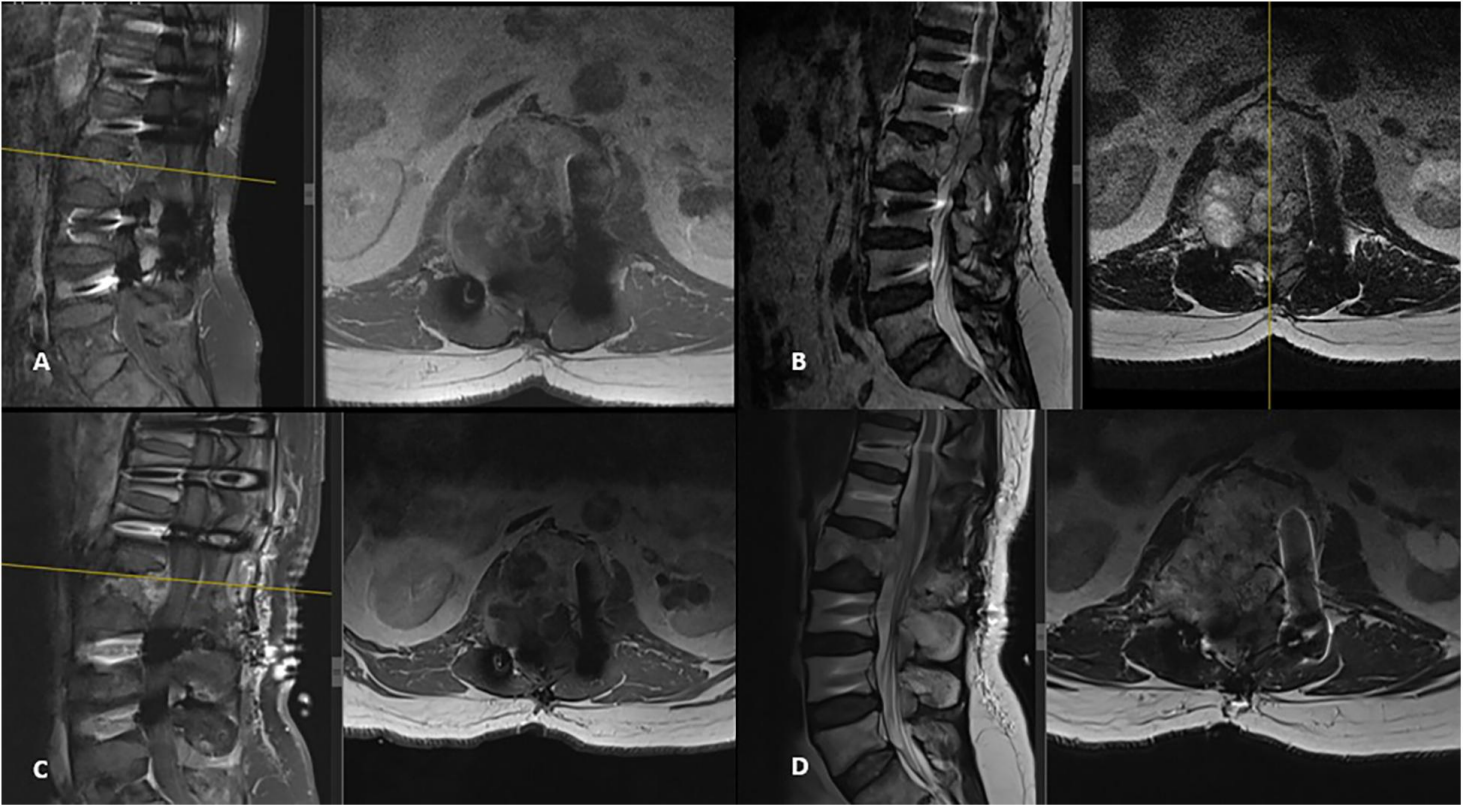
New spinal MRI performed 3 years after the initial surgery revealed a recurrent tumor with progression in the same region. It appeared hypointense on T1-weighted images **(A)** and hyperintense on T2-weighted sagittal and axial images **(B)**, showing diffuse contrast enhancement, spinal canal extension, and dural sac compression. Postoperative T1-weighted **(C)** and T2-weighted **(D)** MRI images demonstrate subtotal tumor removal and adequate decompression.

Three years after the initial operation, the patient presented with complaints of newly developed right-sided sciatica. Neurological examination at this time was unremarkable. A follow-up spinal MRI revealed the progression of the remnant tumor in the same region (Figures 3A,B). The patient underwent a second surgical procedure, during which the tumor was again removed subtotally (Figures 3C,D). As in the previous operation, the tumor was found to be purely extradural. This time, histopathological and immunohistochemical analysis of the specimen confirmed the diagnosis of chordoma. The tumor was predominantly composed of cells with clear or multivacuolated cytoplasm, occasionally forming chordoid and trabecular arrangements within a myxoid stroma. Immunohistochemically, the tumor cells were positive for cytokeratin (CK) AE1/AE3 and negative for S-100, RCC, CK14, CK7, CK20, and PAX8. The MIB-1 labeling index was 10%. Tumor cells showed diffuse positive staining for brachyury, a specific marker for tumors of notochordal origin. The postoperative course was again uneventful, the patients’ symptoms were relieved, and there were no neurological deficits after the surgery. Following the confirmed diagnosis of chordoma, proton beam therapy was administered with a total dose of 66 Gy delivered in 33 fractions. The spinal lesion remained stable over the next 3 years, during which the patient continued to be monitored regularly in the outpatient clinic.

**Figure 4:**
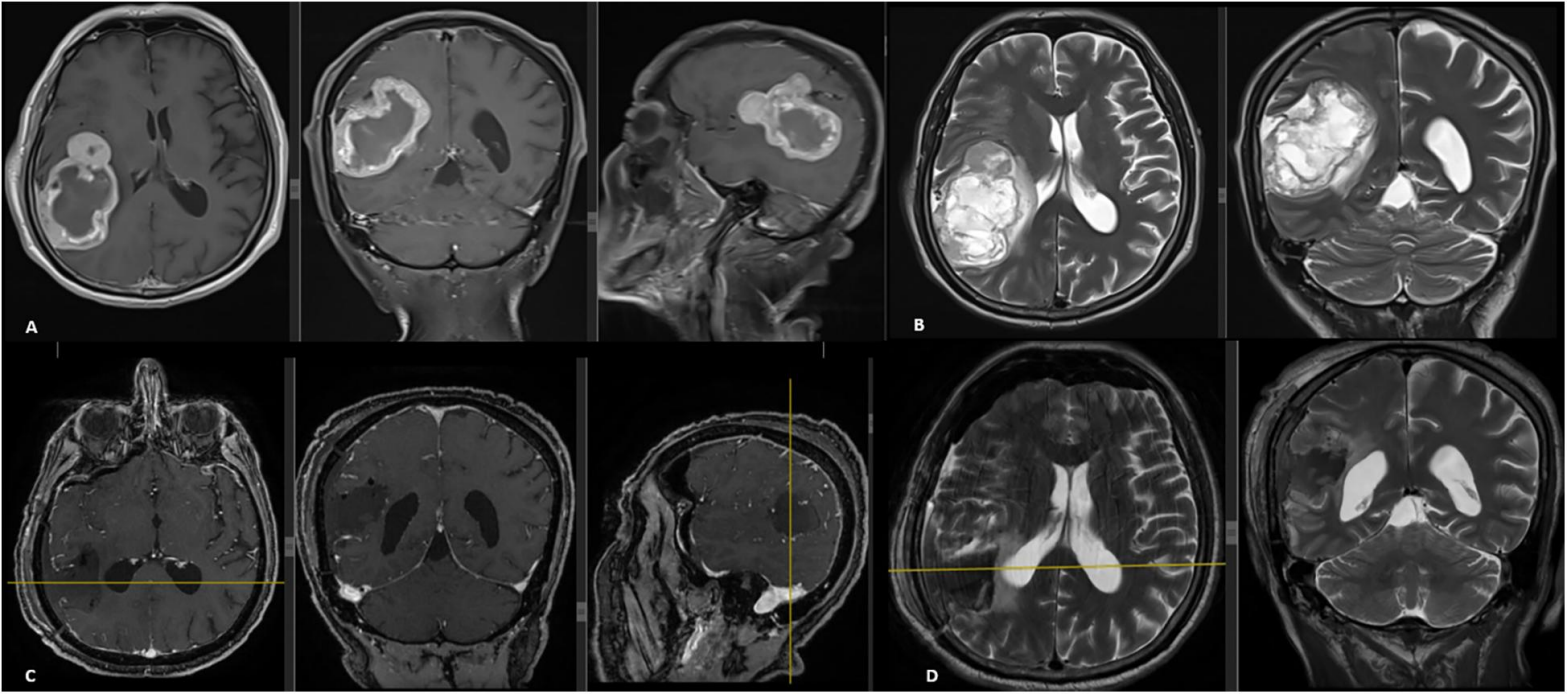
Preoperative cranial MRI **(A,B)** showed a tumoral lesion located in the right frontoparietal region measuring 70 mm × 50 mm × 55 mm, hypointense on T1-weighted and hyperintense on T2-weighted images, with rim-like contrast enhancement. The central areas of the tumor did not enhance with contrast and appeared necrotic and cystic with a hypointense signal. The lesion caused peripheral edema and a 5-mm midline shift. Early postoperative cranial MRI **(C,D)** confirmed total tumor resection.

At his most recent follow-up visit, the patient reported weight loss and newly developed left-sided weakness. He had lost 15 kg over the past 6 months. Neurological examination revealed left-sided hemihypoesthesia, with upper extremity hemiparesis graded at 3/5 and lower extremity hemiparesis at 4/5. Cranial MRI demonstrated a tumoral mass in the right frontoparietal region measuring 70 mm × 50 mm × 55 mm. The lesion appeared hypointense on T1-weighted images and hyperintense on T2-weighted images, with rim-like contrast enhancement. The central portion of the lesion showed no enhancement and appeared necrotic and cystic with hypointense characteristics. The lesion was associated with surrounding edema and caused a 5 mm shift of the midline (Figures 4A,B). Based on these radiological findings and the new clinical presentation of hemiparesis, the lesion was initially considered to be intraaxial, prompting immediate surgical intervention. A neuronavigation-guided right frontoparietal craniotomy was performed. Upon opening the dura, the tumor was identified as extraaxial, with partial attachment to the dura. It was noted to be highly vascularized. Using the tumor's cleavage plane, it was carefully dissected from adjacent neural structures and internally debulked in a stepwise manner. Total resection was achieved with the assistance of neuronavigation and intraoperative ultrasound. The patient was monitored postoperatively in the intensive care unit without any new neurological deficits. Early postoperative MRI confirmed complete removal of the tumor (Figures 4C,D). Following 4 additional days of inpatient care, the patient was discharged from the hospital with no neurological deficits.

Histopathological analysis showed physaliphorous cells with clear or multivacuolated cytoplasm arranged in chordoid, solid, trabecular, and pseudoglandular patterns within a myxoid stroma. The tumor stained positive for CK AE1/AE3, brachyury, and INI-1, with weak S-100 staining observed in a small number of cells. The MIB-1 labeling index was measured at 20%. These findings were consistent with a diagnosis of chordoma. The patient was placed under close clinical surveillance. One year after the surgery, no recurrence was detected, and the patient remains clinically and radiologically stable.

## Discussion

The notochord is a temporary mesodermal structure present during embryonic development, around which the ventral skull base, vertebral column, and sacrum form (2, 10). It first appears during the third week of embryogenesis and begins to regress by approximately the sixth or seventh week (6, 10). In adults, it persists only within the nucleus pulposus of the intervertebral disc (10). Since any remaining notochordal tissue would be expected to reside within bone, chordomas typically develop in extradural locations and are associated with bone destruction (2, 4). Pure intradural intracranial chordomas are exceedingly rare, with only 67 reported cases to date, to the best of our knowledge (1, 2, 6, 11–13).

The exact pathogenesis of intradural chordomas remains unclear. Two main theories have been proposed to explain the development of purely intradural chordomas. The first theory involves ecchordosis physaliphora (EP), which is considered a benign, ectopic remnant of notochordal tissue that usually remains asymptomatic (3, 10, 13). EPs are typically located in the prepontine cistern, range in size from a few millimeters to 2 cm, and are attached to the dorsal clivus via a small pedicle-like structure (2, 10, 13). Their incidence is approximately 2% in autopsy series and 1.7% in MRI studies (10, 13). According to the first theory, the malignant transformation of EP, a benign developmental anomaly, may result in the formation of intradural chordomas (2). This explanation is plausible for tumors arising adjacent to ventral skull base synchondroses, such as the prepontine cistern or intra- or suprasellar regions. However, it does not adequately account for intradural chordomas in less common sites such as the sphenoid wing, anterior cranial fossa, pineal region, or corpus callosum. The second theory proposes that notochordal remnants may become displaced and migrate within the intradural space, potentially as a result of early cranial trauma (2, 11, 14). In our case, the tumor's localization was unusual. It was situated in the right frontoparietal region, distant from the midline and located within the brain parenchyma. Although the patient had a prior history of spinal chordoma, the cranial lesion was not suspected to be a chordoma before surgery. To our knowledge, this specific localization has not been previously reported for intradural chordomas.

We also performed a brief literature review to compile all published cases of intradural chordomas within the cranial vault. Studies involving primary extradural chordomas and spinal chordomas were excluded. In total, 67 cases were identified. The most common locations were the prepontine cistern, with 24 reports covering 32 cases (1, 2, 12), and the intrasellar–suprasellar region, with 16 reports including 20 cases (1, 15). In addition, there were 15 case reports of intradural chordomas in uncommon locations (1, 4, 6, 11, 14, 16–25) (Table 1). Among these 15 cases, only 2 appeared intraaxial on radiological imaging (1, 20). Our current case represents the third case with this radiological characteristic. Previously, only one case reported by Rinaldo et al. (1) demonstrated similar imaging, initially considered a possible glioblastoma before surgery. However, in that instance, the tumor was located in the corpus callosum, a midline structure.

**Table 1:** Reported case reports in the literature of cranial intradural chordomas with uncommon locations.

Author	Publication year	Age/sex	Location	Radiological appearance	Citation
Bulters et al.	2010	24/M	Anterior cranial fossa	Extraaxial	(19)
Dow et al.	2003	9/F	Cerebellar hemisphere	Intraaxial	(20)
Korinth et al.	1999	48/F	Cerebellopontine angle	Extraaxial	(23)
Goodarzi et al.	2017	40/M	Cerebellopontine angle	Extraaxial	(25)
Hazra et al.	2022	70/M	Cerebellopontine angle	Extraaxial	(11)
Rinaldo et al.	2018	69/M	Corpus callosum	Intraaxial	(1)
Katayama et al.	1991	32/F	Foramen magnum	Extraaxial	(22)
Lu et al.	2004	29/F	Meckel's cave	Extraaxial	(24)
Barresi et al.	2012	70/F	Meckel's cave	Extraaxial	(18)
Figueiredo et al.	2011	18/M	Pineal region	Extraaxial	(21)
Kwon et al.	2021	41/M	Pineal region	Extraaxial	(6)
Anderson et al.	2012	13/F	Posterior fossa	Extraaxial	(17)
Kunert et al.	2012	39/M	Sphenoid wing	Extraaxial	(4)
Warnick et al.	1991	58/M	Tentorium cerebelli	Extraaxial	(14)
Commins et al.	1994	51/F	Third ventricle Hypothalamus	Extraaxial	(16)
Current report	2025	67/M	Frontoparietal cortex	Intraaxial	

Histopathological diagnosis of chordoma is based on characteristic cell morphology, including the presence of physaliphorous cells. Immunopositivity for epithelial markers such as CKs and EMA, as well as for the S-100 protein, is also significant (4). Notably, positive staining for brachyury and galectin-3 is especially important for confirming the diagnosis (5). The MIB-1 proliferation index is considered an important prognostic factor, with values above 10.2% associated with tumor recurrence (26). In our case, the absence of S-100 positivity after the first surgery contributed to a misdiagnosis. CKs were also negative, and without brachyury staining, the tumor was mistaken for a metastasis. The histopathological findings suggested clear cell carcinoma, although determining the tumor's origin was difficult. It was only after the second surgery, when brachyury staining was applied, that the correct diagnosis was established. Therefore, we believe that when cells show chordoid and trabecular patterns, additional immunostaining with brachyury and galectin-3, alongside standard S-100, CKs, and EMA staining, may be critical for accurate histopathological diagnosis.

Although the genetics of chordoma have been investigated in both sporadic and familial cases, they are not yet fully understood. In sporadic chordoma cases, aneuploidy has been reported with an average incidence of approximately 53% (27). Genetic research on chordomas indicates that chromosomal instability usually leads to chromosomal gains—especially in regions such as chromosome 7—and losses, particularly in regions 1p and 3p (27, 28). Tumor genetics also offers information regarding prognosis and recurrence, with some evidence suggesting that abnormalities in chromosomes such as 3, 4, 12, 13, and 14 may be more strongly linked to recurrence (27). A study by Yang et al. (29) identified chromosome 7q as a potential locus for familial chordoma, although the previously noted involvement of the 1p locus was not clearly confirmed in their work. The 7q region, important in both familial and sporadic cases, spans about 16 megabases and contains many genes, making it a significant focus for future studies (27, 29). In our patient, there was no family history of chordoma or other tumors, nor were there known genetic or environmental risk factors such as radiation exposure. The case was considered sporadic. Genetic testing was not performed before diagnosis, but the tumor tissue was stored in a biobank for possible future analysis.

The radiological characteristics of chordomas are well established; however, the challenge lies in including intradural chordomas in the differential diagnosis when they appear in unexpected locations, as in our case. Chordomas typically appear as hypointense lesions on T1-weighted images and hyperintense on T2-weighted images, with contrast enhancement (9). Poorly differentiated histopathology cases have shown diffusion restriction and low apparent diffusion coefficient signals (2). The spinal tumor in our case exhibited classic chordoma features, including bone destruction, but these features are also common in metastatic tumors, which contributed to the histopathological misdiagnosis after the first surgery. The intracranial intradural chordoma also appeared hypointense on T1-weighted and hyperintense on T2-weighted images, with peripheral rim-like contrast enhancement. Although these radiological findings align with chordoma, its unusual location within the brain parenchyma—rather than near the midline—prompted consideration of other possible diagnoses. The tumor was initially thought to be intraaxial before surgery, and its extraaxial nature was only discovered during the operation.

Chordomas can metastasize, but this typically occurs in the advanced stages of the disease (30). The lungs are the most common site of metastasis, with younger patients being more frequently affected (8). Other potential metastatic sites include the liver, lymph nodes, bones, and cerebrospinal fluid dissemination (8, 31). Steenberghs et al. (32) and Badwal et al. (33) have reported spinal and meningeal metastases originating from intradural chordomas. Although intradural chordomas are considered to have a better prognosis (3, 6), they may still tend to metastasize (30). Cases of seeding metastases from cranial chordomas have been reported previously (8, 30). Surgery for intracranial chordomas has been suggested as a possible factor contributing to surgical seeding metastasis (30). However, this remains speculative, as no published cases include spinal MRI at the time of diagnosis and/or surgery for intracranial intradural chordomas (7). Kawanabe et al. reported the only case demonstrating spontaneous coexistence of spinal and cranial intradural chordomas in the same patient (7). Our case represents an exceptionally rare example compared to those reported in the literature. In this case, a classical spinal chordoma was diagnosed and surgically treated. At the time of diagnosis, a PET/CT scan showed no intracranial lesions. Six years after the initial surgery, the patient developed an intracranial mass. It is difficult to definitively classify this lesion as a metastasis from the spinal chordoma, but prior imaging clearly demonstrated that the intracranial lesion was not present at the time of the initial spinal chordoma diagnosis. Another important point is that the dura was not opened during either of the two spinal surgeries, meaning the spinal chordoma was located extradurally. It is also possible to speculate that a small EP-type lesion may have migrated to the brain parenchyma during embryonic development and remained dormant for years before differentiating into an intradural chordoma. It is possible that this lesion may have remained undetected in PET/CT scans. Although the mechanism is difficult to explain, to consider the current lesion as a metastasis occurring in the opposite direction than typically expected—that is, spreading from the spinal column to the cranial vault, is also possible.

Regardless of the mechanism of occurrence, our case is unique because of its parenchymal location, which is distant from the midline and skull base—typical sites for chordomas. Despite the patients’ known history, the tumor's radiological features and location led us to consider diagnoses other than chordoma before pathological examination. Further research is needed to explore potential metastatic mechanisms or the coexistence of dual tumors in different locations during the clinical course of chordomas.

## Data Availability

The raw data supporting the conclusions of this article will be made available by the authors, without undue reservation.

## References

[1] Rinaldo L Priemer DS Vortmeyer AO Cohen-Gadol AA Brat DJ Mahajan A Chordoma of the corpus callosum: case report. J Neurosurg. (2018) 131(5):1380–6. 10.3171/2018.6.JNS181028 30497142

[2] de Almeida GB Januário G Carvalho R. Nonenhancing intracranial intradural chordoma mimicking an epidermoid cyst on magnetic resonance imaging: a case report. Radiol Case Rep. (2021) 16(8):2306–10. 10.1016/j.radcr.2021.05.057 34194595 PMC8233103

[3] AlOtaibi F Guiot MC Muanza T Di Maio S. Giant petroclival primary intradural chordoma: case report and systematic review of the literature. J Neurol Surg Rep. (2014) 75(1):e160–9. 10.1055/s-0034-1378157 25083378 PMC4110134

[4] Kunert P Dziedzic T Matyja E Marchel A. Intradural chordoma mimicking a lateral sphenoid wing meningioma: a case report. Folia Neuropathol. (2012) 50(4):407–12. 10.5114/fn.2012.32375 23319197

[5] Vellutini Ede A de Oliveira MF. Intradural chordoma presenting with intratumoral bleeding. J Clin Neurosci. (2016) 25:139–42. 10.1016/j.jocn.2015.07.023 26563604

[6] Kwon JE So Young J Hwang K Lee KS Choe G Kim CY Management challenges associated with a pineal region chordoma: illustrative case. J Neurosurg Case Lessons. (2021) 1(24):CASE21110. 10.3171/CASE21110 35855099 PMC9245841

[7] Kawanabe Y Ueda S Sasaki N Hoshimaru M. Simultaneous discovery of cranial and spinal intradural chordomas: case report. Neurol Med Chir. (2014) 54(11):930–5. 10.2176/nmc.cr.2013-0150 PMC4533341 24477062

[8] Liu X Li M Chen G. Intradural spinal seeding metastasis of clival chordoma: a case report. Transl Cancer Res. (2022) 11(9):3426–33. 10.21037/tcr-22-211 36237273 PMC9552250

[9] George B Bresson D Herman P Froelich S. Chordomas: a review. Neurosurg Clin N Am. (2015) 26(3):437–52. 10.1016/j.nec.2015.03.012 26141362

[10] Bhat DI Yasha M Rojin A Sampath S Shankar SK. Intradural clival chordoma: a rare pathological entity. J Neurooncol. (2010) 96(2):287–90. 10.1007/s11060-009-9949-6 19575150

[11] Hazra D Balasubramanian C Das S Tiwari M Ghosh A. A rare case report of an intradural left cerebellopontine angle chordoma. Asian J Neurosurg. (2022) 17(1):134–6. 10.1055/s-0042-1749128. eCollection 2022 Mar. 35873840 PMC9298575

[12] Ito N Fujii H Kuroda R Matsuki M Mori H. Intradural chordoma mimicking an epidural cyst on imaging. Cureus. (2023) 15(6):e40610. 10.7759/cureus.40610. eCollection 2023 Jun. 37476114 PMC10354377

[13] Vinke RS Lamers EC Kusters B van Lindert EJ. Intradural prepontine chordoma in an 11-year-old boy. A case report. Childs Nerv Syst. (2016) 32(1):169–73. 10.1007/s00381-015-2818-z 26216058 PMC4735251

[14] Warnick RE Raisanen J Kaczmar T Jr Davis RL Prados MD. Intradural chordoma of the tentorium cerebelli. Case report. J Neurosurg. (1991) 74(3):508–11. 10.3171/jns.1991.74.3.0508 1993918

[15] Li B Kim MG Dominguez JF Feldstein E Kleinmann G Al-Mufti F Intrasellar hemorrhagic chordoma masquerading as pituitary apoplexy: case report and review of literature. Br J Neurosurg. (2023) 37(6):1685–8. 10.1080/02688697.2021.1941761 34148480

[16] Commins D Baran GA Molleston M Vollmer D. Hypothalamic chordoma. Case report. J Neurosurg. (1994) 81:130–2. 10.3171/jns.1994.81.1.0130 8207515

[17] Anderson S Sato Y Kirby P Buatti JM Menezes A. Disseminated subarachnoid chordoma: long-term favorable follow-up of a pediatric patient. Pediatr Radiol. (2012) 42:878–80. 10.1007/s00247-011-2266-1 21984317

[18] Barresi V Caffo M Alafaci C Granata F Tuccari G. Intradural chordoma of the Meckel’s cave: a challenging differential diagnosis. Neuropathology. (2012) 32:577–82. 10.1111/j.1440-1789.2011.01295.x 22260529

[19] Bulters DO Webb A Shenouda E. Chordoma of the anterior skull base presenting as a swelling of the medial canthus of the eye. Br J Oral Maxillofac Surg. (2010) 48:211–3. 10.1016/j.bjoms.2009.07.023 19733943

[20] Dow GR Robson DK Jaspan T Punt JA. Intradural cerebellar chordoma in a child: a case report and review of the literature. Childs Nerv Syst. (2003) 19:188–91. 10.1007/s00381-002-0707-8 12644872

[21] Figueiredo EG Tavares WM Welling L Rosemberg S Teixeira MJ. Ectopic pineal chordoma. Surg Neurol Int. (2011) 2:145. 10.4103/2152-7806.85986 22059140 PMC3205502

[22] Katayama Y Tsubokawa T Hirasawa T Takahata T Nemoto N. Intradural extraosseous chordoma in the foramen magnum region. Case report. J Neurosurg. (1991) 75:976–9. 10.3171/jns.1991.75.6.0976 1941129

[23] Korinth M Schönrock L Mayfrank L Gilsbach JM. Primary intradural pontocerebellar chordoma metastasizing in the subarachnoid spinal canal. Zentralbl Neurochir. (1999) 60:146–50. PMID: 10726338

[24] Lu CY Chai CY Chiang IC. Chordoma mimicking the trigeminal schwannoma: a case report. Clin Imaging. (2004) 28:187–90. 10.1016/S0899-7071(03)00236-5 15158222

[25] Goodarzi A Toussi A Lechpammer M Lee D Shahlaie K. Intradural chordoma of cerebellopontine angle: case report and review. World Neurosurg. (2017) 107:1052.e11–6. 10.1016/j.wneu.2017.08.124 28866065

[26] Ito E Saito K Nagatani T Ishiyama J Terada K Yoshida M Intradural cranial chordoma. World Neurosurg. (2010) 73(3):194–7. discussion e31. 10.1016/j.surneu.2009.01.003 20860957

[27] Yakkioui Y van Overbeeke JJ Santegoeds R van Engeland M Temel Y. Chordoma: the entity. Biochim Biophys Acta. (2005) 1846(2):655–69. 10.1016/j.bbcan.2014.07.012 25193090

[28] Bayrakli F Guney I Kilic T Ozek M Pamir MN. New candidate chromosomal regions for chordoma development. Surg Neurol. (2007) 68:425–30. 10.1016/j.surneu.2006.11.046 17714767

[29] Yang X Beerman M Bergen AW Parry DM Sheridan E Liebsch NJ Corroboration of a familial chordoma locus on chromosome 7q and evidence of genetic heterogeneity using single nucleotide polymorphisms (SNPs). Int J Cancer. (2005) 116:487–91. 10.1002/ijc.21006 15818627

[30] Zhang J Gao CP Liu XJ Xu WJ. Intradural cervical chordoma with diffuse spinal leptomeningeal spread: case report and review of the literature. Eur Spine J. (2018) 27(3):440–5. 10.1007/s00586-017-5443-6 29313091

[31] Walcott BP Nahed BV Mohyeldin A Coumans JV Kahle KT Ferreira MJ. Chordoma: current concepts, management, and future directions. Lancet Oncol. (2012) 13(2):e69–76. 10.1016/S1470-2045(11)70337-0 22300861

[32] Steenberghs J Kiekens C Menten J Monstrey J. Intradural chordoma without bone involvement. Case report and review of the literature. J Neurosurg. (2002) 97(1):94–7. 10.3171/spi.2002.97.1.1 12120659

[33] Badwal S Pal L Basu A Saxena S. Multiple synchronous spinal extra-osseous intradural chordomas: is it a distinct entity? Br J Neurosurg. (2006) 20(2):99–103. 10.1080/02688690600682614 16753627

